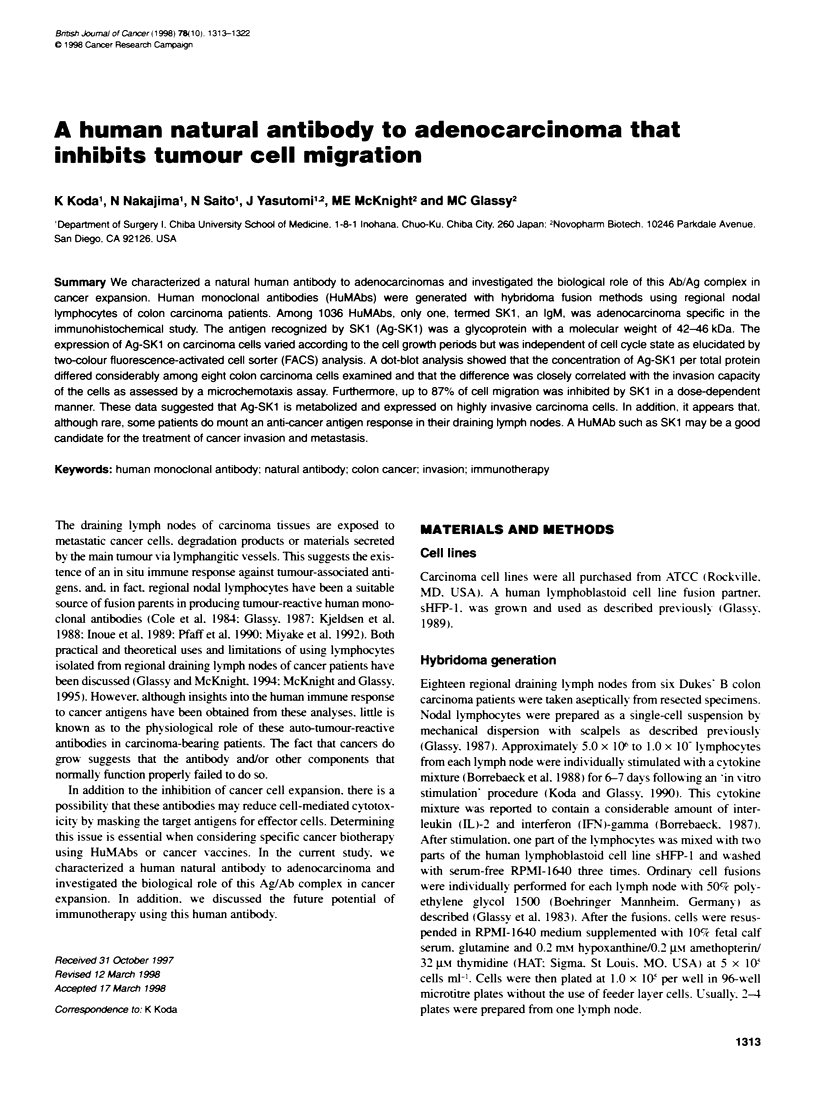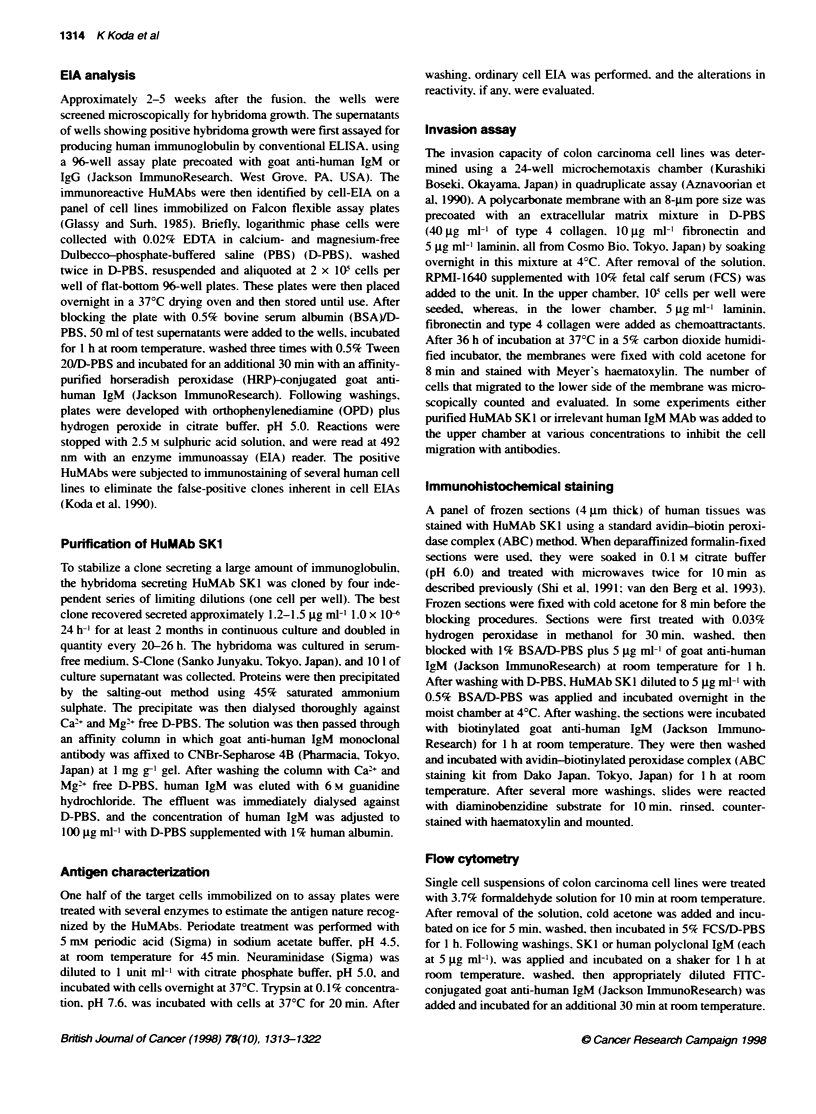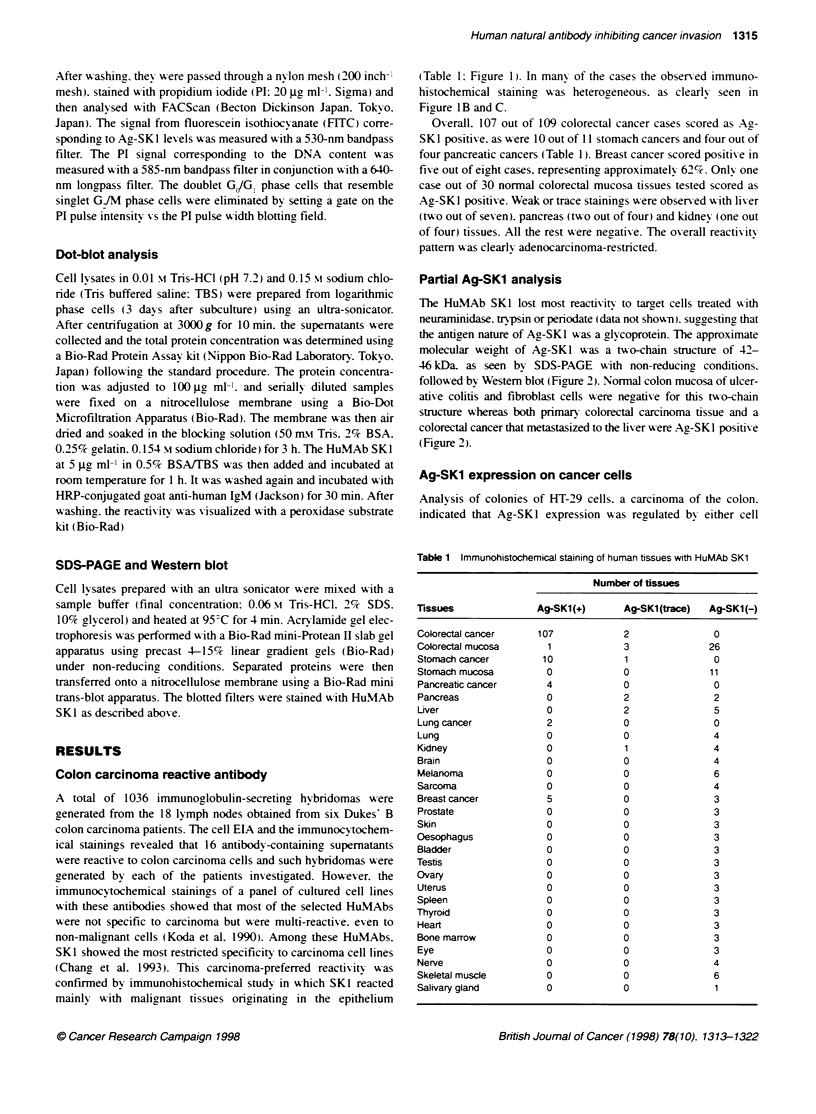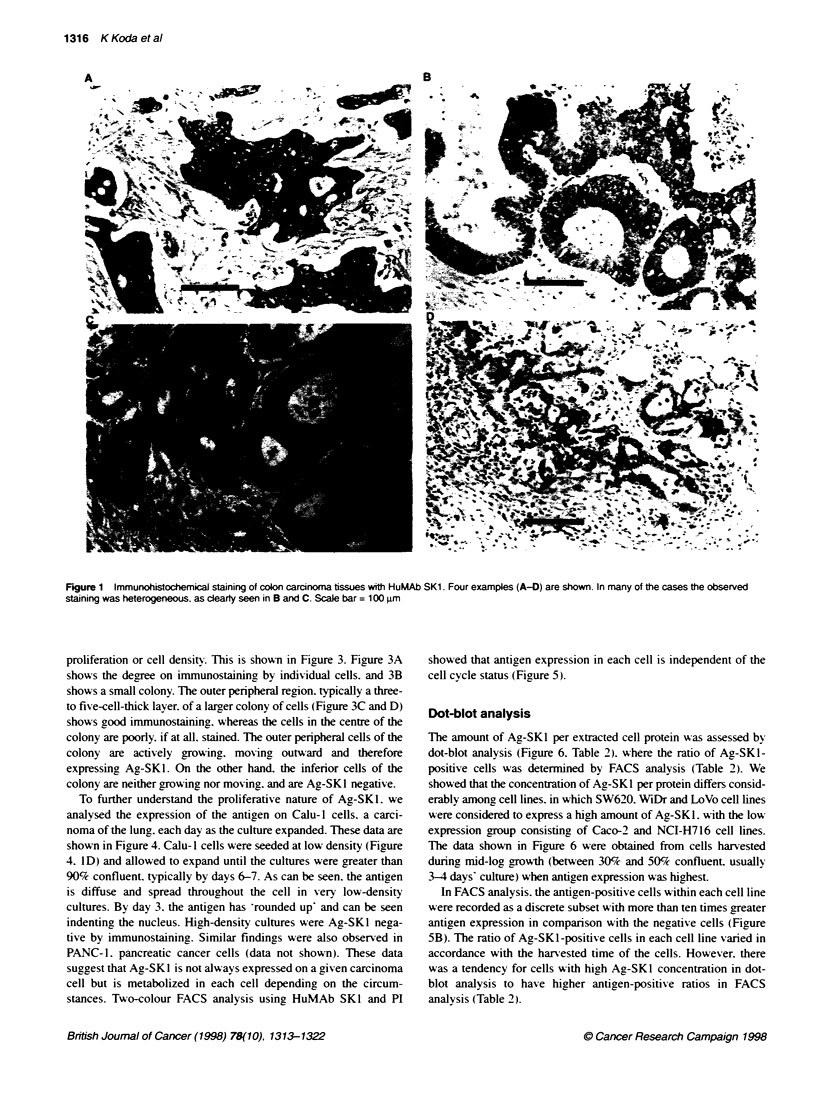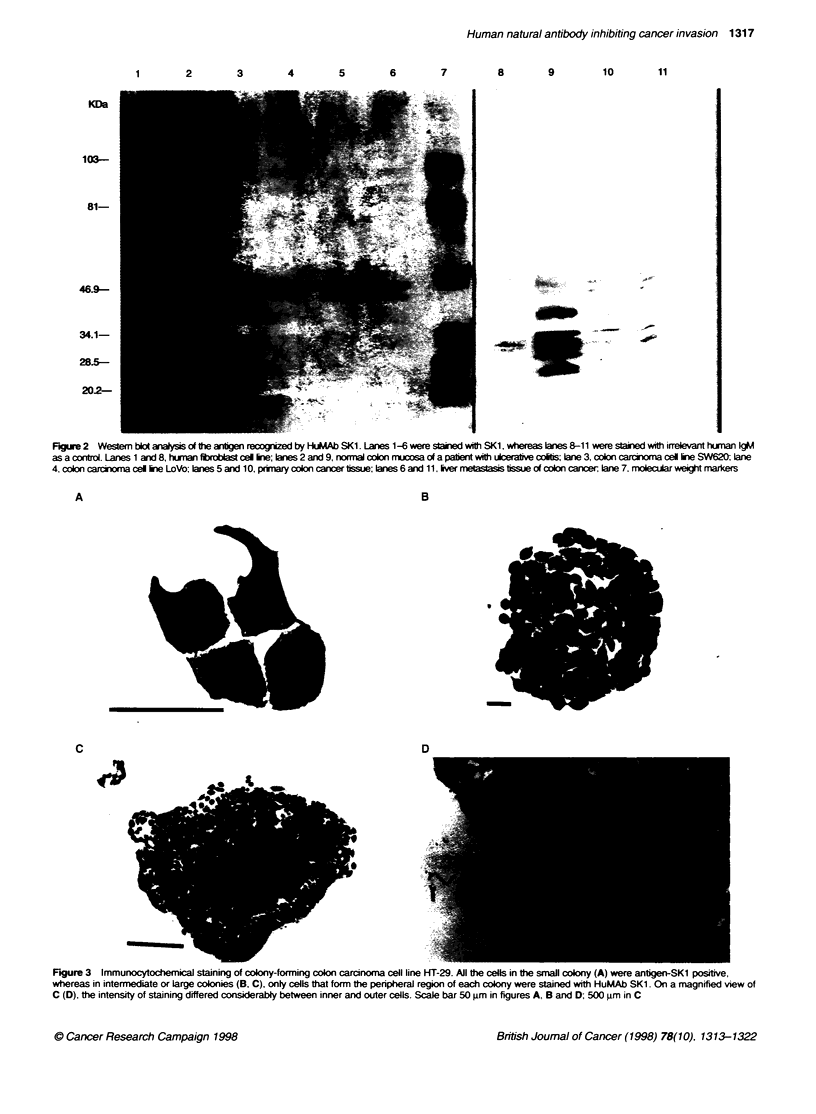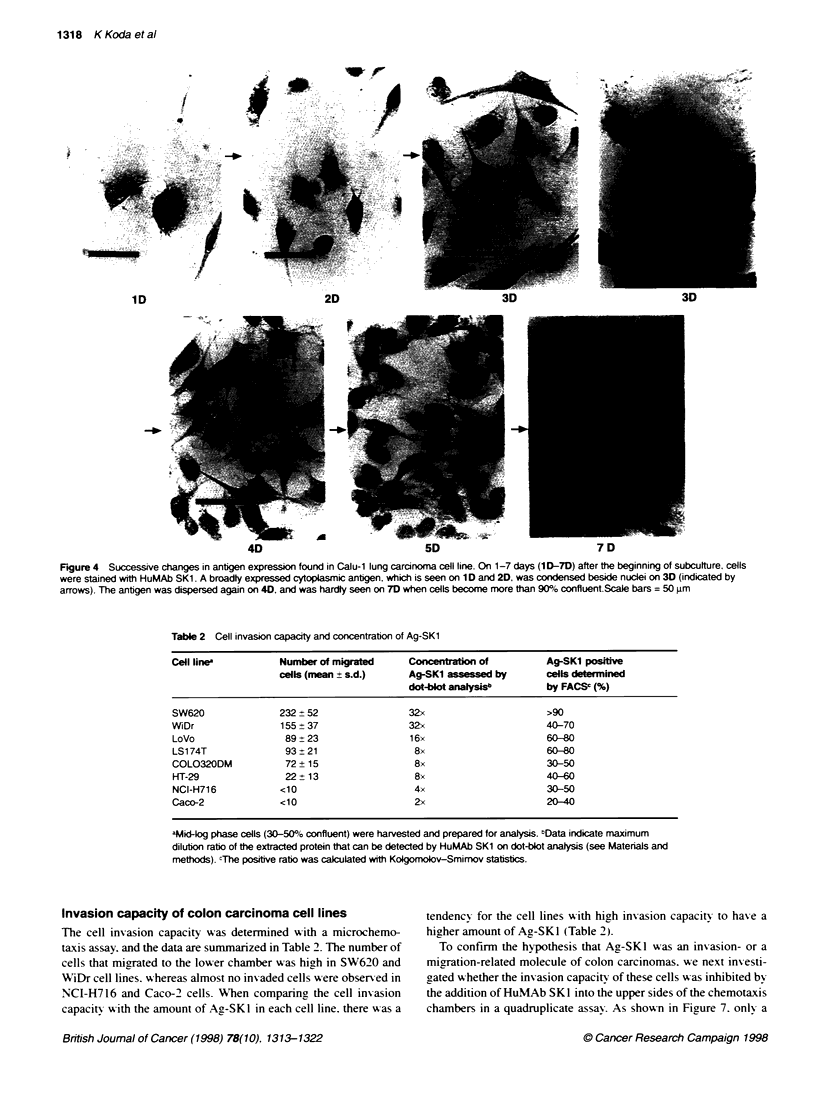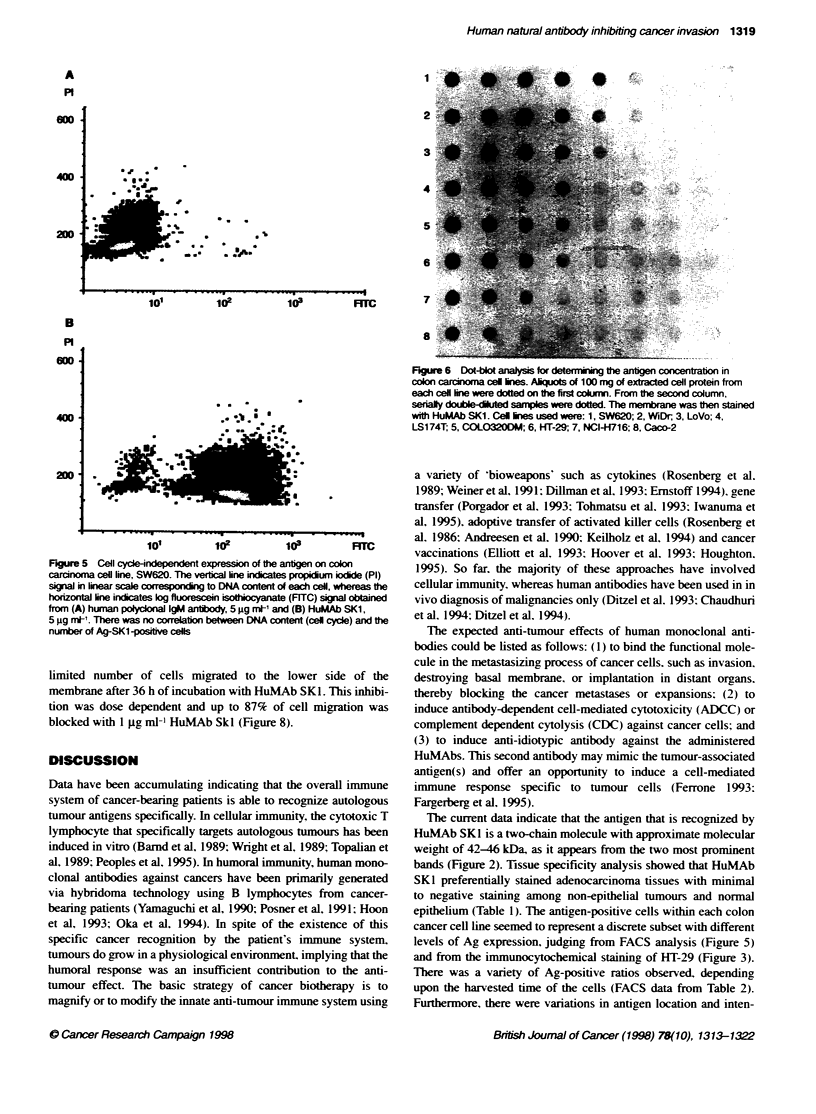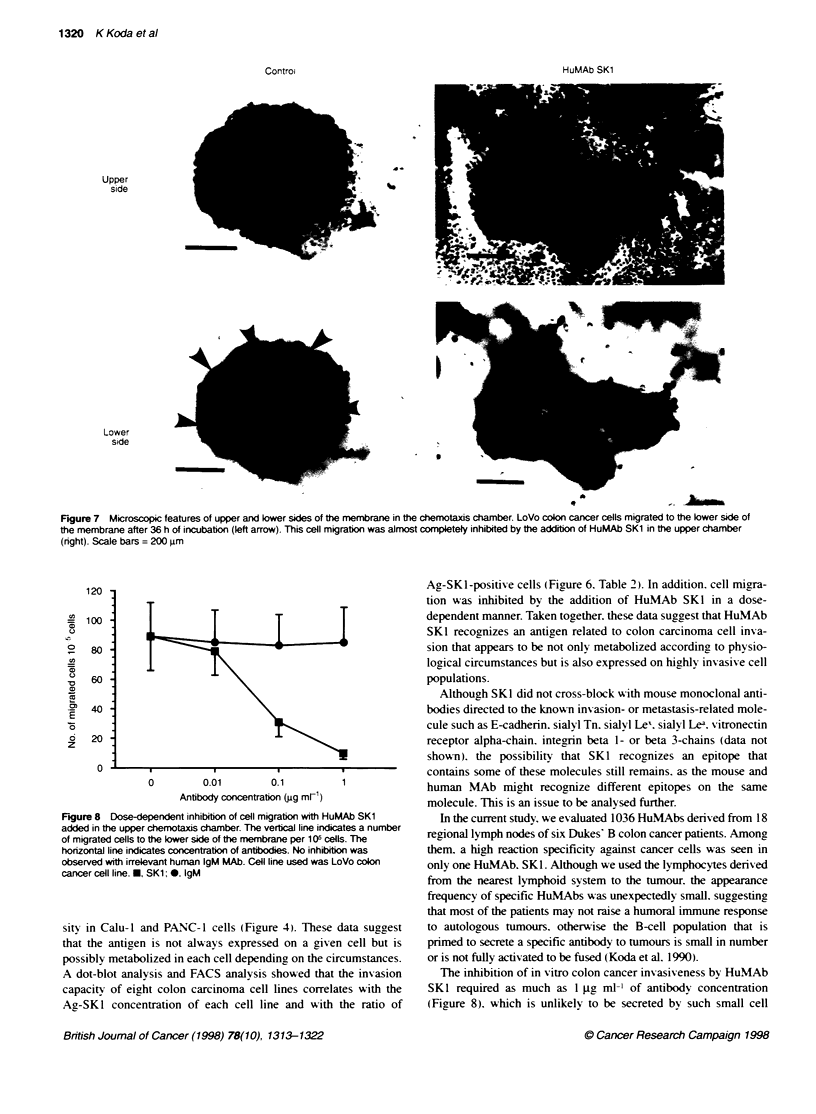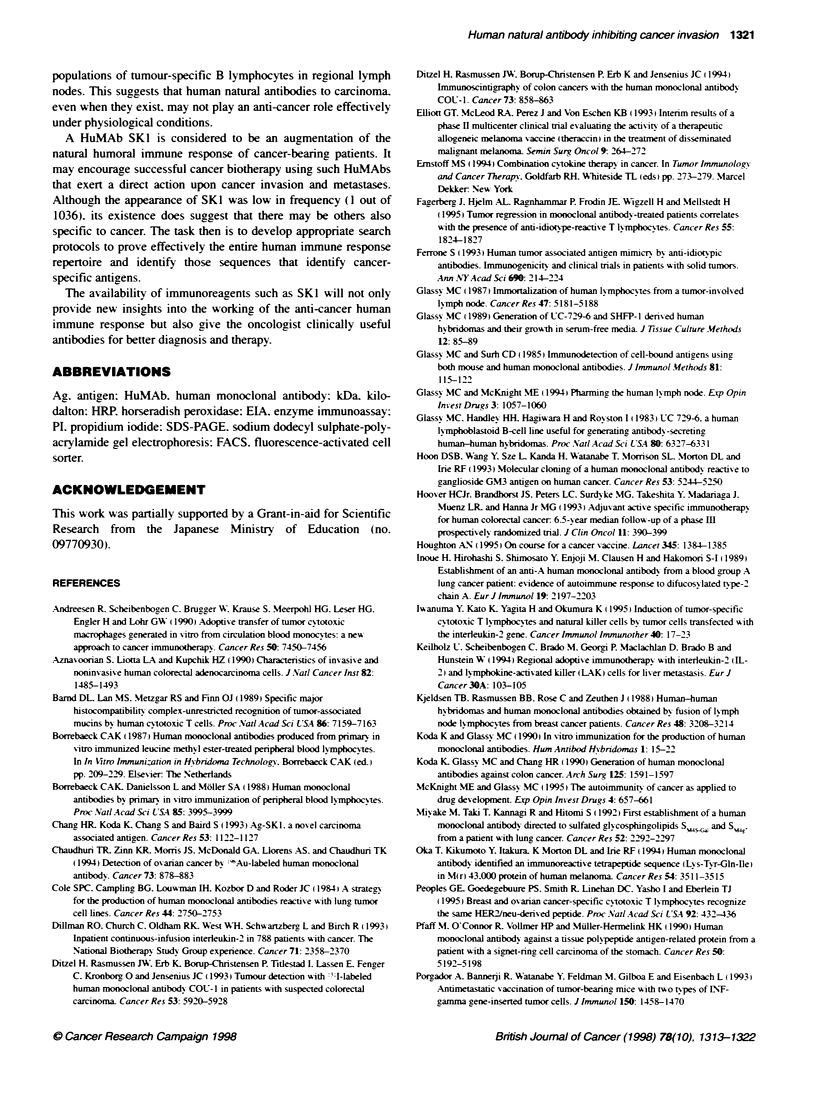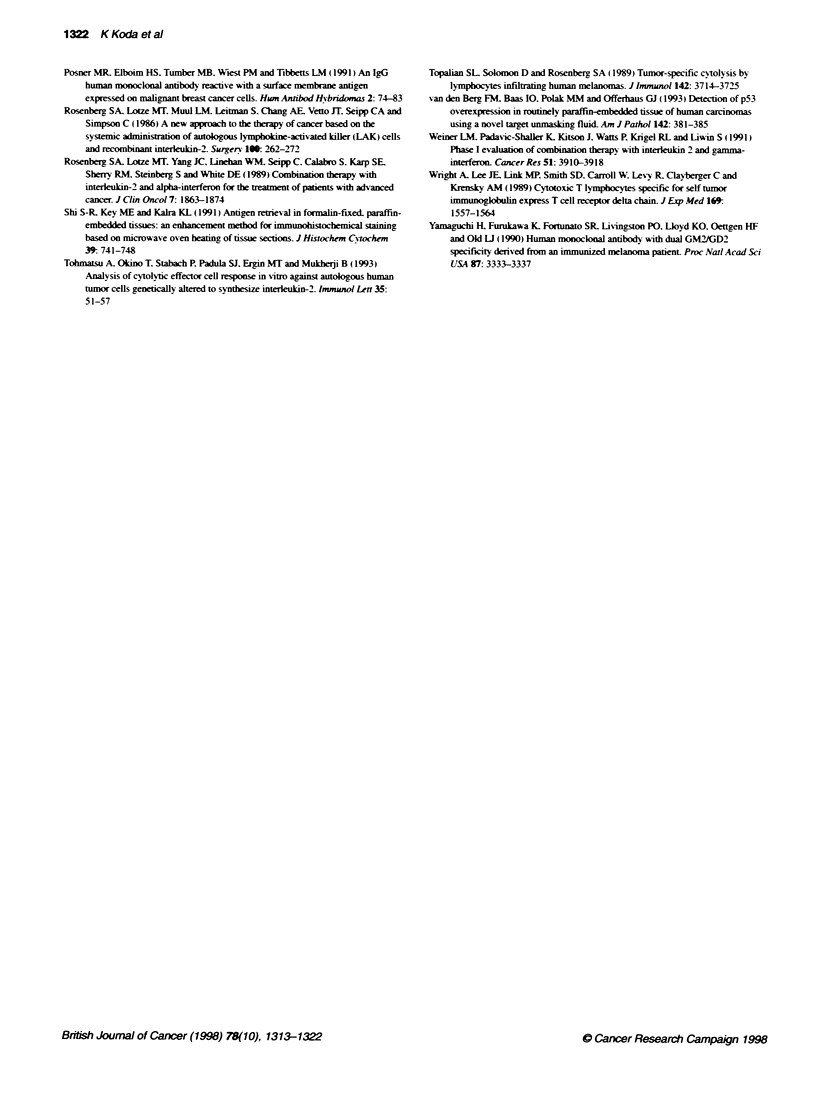# A human natural antibody to adenocarcinoma that inhibits tumour cell migration.

**DOI:** 10.1038/bjc.1998.677

**Published:** 1998-11

**Authors:** K. Koda, N. Nakajima, N. Saito, J. Yasutomi, M. E. McKnight, M. C. Glassy

**Affiliations:** Department of Surgery I, Chiba University School of Medicine, Chiba City, Japan.

## Abstract

**Images:**


British Journal of Cancer (1998) 78(10). 1313–1322
© 1998 Cancer Research Campaign

### A human natural antibody to adenocarcinoma that inhibits tumour cell migration

**K Koda[1], N Nakajima[1], N Saito[1], J Yasutomi[1,2], ME McKnight[2] and MC Glassy[2]**

[1]Department of Surgery I. Chiba University School of Medicine. 1-8-1 Inohana. Chuo-Ku. Chiba City. 260 Japan: [2]Novopharm Biotech. 10246 Parkdale Avenue. San Diego. CA 92126. USA

**Summary** We characterized a natural human antibody to adenocarcinomas and investigated the biological role of this Ab/Ag complex in cancer expansion. Human monoclonal antibodies (HuMAbs) were generated with hybridoma fusion methods using regional nodal lymphocytes of colon carcinoma patients. Among 1036 HuMAbs, only one, termed SK1, an IgM, was adenocarcinoma specific in the immunohistochemical study. The antigen recognized by SK1 (Ag-SK1) was a glycoprotein with a molecular weight of 42–46 kDa. The expression of Ag-SK1 on carcinoma cells varied according to the cell growth periods but was independent of cell cycle state as elucidated by two-colour fluorescence-activated cell sorter (FACS) analysis. A dot-blot analysis showed that the concentration of Ag-SK1 per total protein differed considerably among eight colon carcinoma cells examined and that the difference was closely correlated with the invasion capacity of the cells as assessed by a microchemotaxis assay. Furthermore, up to 87% of cell migration was inhibited by SK1 in a dose-dependent manner. These data suggested that Ag-SK1 is metabolized and expressed on highly invasive carcinoma cells. In addition, it appears that, although rare, some patients do mount an anti-cancer antigen response in their draining lymph nodes. A HuMAb such as SK1 may be a good candidate for the treatment of cancer invasion and metastasis.

**Keywords:** human monoclonal antibody; natural antibody; colon cancer; invasion; immunotherapy

The draining lymph nodes of carcinoma tissues are exposed to metastatic cancer cells, degradation products or materials secreted by the main tumour via lymphangitic vessels. This suggests the existence of an in situ immune response against tumour-associated antigens, and, in fact, regional nodal lymphocytes have been a suitable source of fusion parents in producing tumour-reactive human monoclonal antibodies (Cole et al, 1984; Glassy, 1987; Kjeldsen et al, 1988; Inoue et al, 1989; Pfaff et al, 1990; Miyake et al, 1992). Both practical and theoretical uses and limitations of using lymphocytes isolated from regional draining lymph nodes of cancer patients have been discussed (Glassy and McKnight, 1994; McKnight and Glassy, 1995). However, although insights into the human immune response to cancer antigens have been obtained from these analyses, little is known as to the physiological role of these auto-tumour-reactive antibodies in carcinoma-bearing patients. The fact that cancers do grow suggests that the antibody and/or other components that normally function properly failed to do so.

In addition to the inhibition of cancer cell expansion, there is a possibility that these antibodies may reduce cell-mediated cytotoxicity by masking the target antigens for effector cells. Determining this issue is essential when considering specific cancer biotherapy using HuMAbs or cancer vaccines. In the current study, we characterized a human natural antibody to adenocarcinoma and investigated the biological role of this Ag/Ab complex in cancer expansion. In addition, we discussed the future potential of immunotherapy using this human antibody.

*Received 31 October 1997*
*Revised 12 March 1998*
*Accepted 17 March 1998*

*Correspondence to:* K Koda

### MATERIALS AND METHODS

### Cell lines

Carcinoma cell lines were all purchased from ATCC (Rockville, MD, USA). A human lymphoblastoid cell line fusion partner, sHFP-1, was grown and used as described previously (Glassy, 1989).

### Hybridoma generation

Eighteen regional draining lymph nodes from six Dukes' B colon carcinoma patients were taken aseptically from resected specimens. Nodal lymphocytes were prepared as a single-cell suspension by mechanical dispersion with scalpels as described previously (Glassy, 1987). Approximately $5.0 \times 10^6$ to $1.0 \times 10^7$ lymphocytes from each lymph node were individually stimulated with a cytokine mixture (Borrebaeck et al, 1988) for 6–7 days following an 'in vitro stimulation' procedure (Koda and Glassy, 1990). This cytokine mixture was reported to contain a considerable amount of interleukin (IL)-2 and interferon (IFN)-gamma (Borrebaeck, 1987). After stimulation, one part of the lymphocytes was mixed with two parts of the human lymphoblastoid cell line sHFP-1 and washed with serum-free RPMI-1640 three times. Ordinary cell fusions were individually performed for each lymph node with 50% polyethylene glycol 1500 (Boehringer Mannheim, Germany) as described (Glassy et al, 1983). After the fusions, cells were resuspended in RPMI-1640 medium supplemented with 10% fetal calf serum, glutamine and 0.2 mM hypoxanthine/0.2 μM amethopterin/32 μM thymidine (HAT; Sigma, St Louis, MO, USA) at $5 \times 10^5$ cells $ml^{-1}$. Cells were then plated at $1.0 \times 10^5$ per well in 96-well microtitre plates without the use of feeder layer cells. Usually, 2–4 plates were prepared from one lymph node.

### EIA analysis

Approximately 2–5 weeks after the fusion, the wells were screened microscopically for hybridoma growth. The supernatants of wells showing positive hybridoma growth were first assayed for producing human immunoglobulin by conventional ELISA, using a 96-well assay plate precoated with goat anti-human IgM or IgG (Jackson ImmunoResearch, West Grove, PA, USA). The immunoreactive HuMAbs were then identified by cell-EIA on a panel of cell lines immobilized on Falcon flexible assay plates (Glassy and Surh, 1985). Briefly, logarithmic phase cells were collected with 0.02% EDTA in calcium- and magnesium-free Dulbecco–phosphate-buffered saline (PBS) (D-PBS), washed twice in D-PBS, resuspended and aliquoted at $2 \times 10^5$ cells per well of flat-bottom 96-well plates. These plates were then placed overnight in a 37°C drying oven and then stored until use. After blocking the plate with 0.5% bovine serum albumin (BSA)/D-PBS, 50 ml of test supernatants were added to the wells, incubated for 1 h at room temperature, washed three times with 0.5% Tween 20/D-PBS and incubated for an additional 30 min with an affinity-purified horseradish peroxidase (HRP)-conjugated goat anti-human IgM (Jackson ImmunoResearch). Following washings, plates were developed with orthophenylenediamine (OPD) plus hydrogen peroxide in citrate buffer, pH 5.0. Reactions were stopped with 2.5 M sulphuric acid solution, and were read at 492 nm with an enzyme immunoassay (EIA) reader. The positive HuMAbs were subjected to immunostaining of several human cell lines to eliminate the false-positive clones inherent in cell EIAs (Koda et al, 1990).

### Purification of HuMAb SK1

To stabilize a clone secreting a large amount of immunoglobulin, the hybridoma secreting HuMAb SK1 was cloned by four independent series of limiting dilutions (one cell per well). The best clone recovered secreted approximately 1.2–1.5 $\mu g\ ml^{-1}$ $1.0 \times 10^{-6}$ 24 $h^{-1}$ for at least 2 months in continuous culture and doubled in quantity every 20–26 h. The hybridoma was cultured in serum-free medium, S-Clone (Sanko Junyaku, Tokyo, Japan), and 10 l of culture supernatant was collected. Proteins were then precipitated by the salting-out method using 45% saturated ammonium sulphate. The precipitate was then dialysed thoroughly against $Ca^{2+}$ and $Mg^{2+}$ free D-PBS. The solution was then passed through an affinity column in which goat anti-human IgM monoclonal antibody was affixed to CNBr-Sepharose 4B (Pharmacia, Tokyo, Japan) at 1 mg $g^{-1}$ gel. After washing the column with $Ca^{2+}$ and $Mg^{2+}$ free D-PBS, human IgM was eluted with 6 M guanidine hydrochloride. The effluent was immediately dialysed against D-PBS, and the concentration of human IgM was adjusted to 100 $\mu g\ ml^{-1}$ with D-PBS supplemented with 1% human albumin.

### Antigen characterization

One half of the target cells immobilized on to assay plates were treated with several enzymes to estimate the antigen nature recognized by the HuMAbs. Periodate treatment was performed with 5 mM periodic acid (Sigma) in sodium acetate buffer, pH 4.5, at room temperature for 45 min. Neuraminidase (Sigma) was diluted to 1 unit $ml^{-1}$ with citrate phosphate buffer, pH 5.0, and incubated with cells overnight at 37°C. Trypsin at 0.1% concentration, pH 7.6, was incubated with cells at 37°C for 20 min. After washing, ordinary cell EIA was performed, and the alterations in reactivity, if any, were evaluated.

### Invasion assay

The invasion capacity of colon carcinoma cell lines was determined using a 24-well microchemotaxis chamber (Kurashiki Boseki, Okayama, Japan) in quadruplicate assay (Aznavoorian et al, 1990). A polycarbonate membrane with an 8-μm pore size was precoated with an extracellular matrix mixture in D-PBS (40 $\mu g\ ml^{-1}$ of type 4 collagen, 10 $\mu g\ ml^{-1}$ fibronectin and 5 $\mu g\ ml^{-1}$ laminin, all from Cosmo Bio, Tokyo, Japan) by soaking overnight in this mixture at 4°C. After removal of the solution, RPMI-1640 supplemented with 10% fetal calf serum (FCS) was added to the unit. In the upper chamber, $10^5$ cells per well were seeded, whereas, in the lower chamber, 5 $\mu g\ ml^{-1}$ laminin, fibronectin and type 4 collagen were added as chemoattractants. After 36 h of incubation at 37°C in a 5% carbon dioxide humidified incubator, the membranes were fixed with cold acetone for 8 min and stained with Meyer's haematoxylin. The number of cells that migrated to the lower side of the membrane was microscopically counted and evaluated. In some experiments either purified HuMAb SK1 or irrelevant human IgM MAb was added to the upper chamber at various concentrations to inhibit the cell migration with antibodies.

### Immunohistochemical staining

A panel of frozen sections (4 μm thick) of human tissues was stained with HuMAb SK1 using a standard avidin–biotin peroxidase complex (ABC) method. When deparaffinized formalin-fixed sections were used, they were soaked in 0.1 M citrate buffer (pH 6.0) and treated with microwaves twice for 10 min as described previously (Shi et al, 1991; van den Berg et al, 1993). Frozen sections were fixed with cold acetone for 8 min before the blocking procedures. Sections were first treated with 0.03% hydrogen peroxidase in methanol for 30 min, washed, then blocked with 1% BSA/D-PBS plus 5 $\mu g\ ml^{-1}$ of goat anti-human IgM (Jackson ImmunoResearch) at room temperature for 1 h. After washing with D-PBS, HuMAb SK1 diluted to 5 $\mu g\ ml^{-1}$ with 0.5% BSA/D-PBS was applied and incubated overnight in the moist chamber at 4°C. After washing, the sections were incubated with biotinylated goat anti-human IgM (Jackson ImmunoResearch) for 1 h at room temperature. They were then washed and incubated with avidin–biotinylated peroxidase complex (ABC staining kit from Dako Japan, Tokyo, Japan) for 1 h at room temperature. After several more washings, slides were reacted with diaminobenzidine substrate for 10 min, rinsed, counterstained with haematoxylin and mounted.

### Flow cytometry

Single cell suspensions of colon carcinoma cell lines were treated with 3.7% formaldehyde solution for 10 min at room temperature. After removal of the solution, cold acetone was added and incubated on ice for 5 min, washed, then incubated in 5% FCS/D-PBS for 1 h. Following washings, SK1 or human polyclonal IgM (each at 5 $\mu g\ ml^{-1}$), was applied and incubated on a shaker for 1 h at room temperature, washed, then appropriately diluted FITC-conjugated goat anti-human IgM (Jackson ImmunoResearch) was added and incubated for an additional 30 min at room temperature.

© Cancer Research Campaign 1998

After washing, they were passed through a nylon mesh (200 inch$^{-1}$ mesh), stained with propidium iodide (PI; 20 μg ml$^{-1}$, Sigma) and then analysed with FACScan (Becton Dickinson Japan, Tokyo, Japan). The signal from fluorescein isothiocyanate (FITC) corresponding to Ag-SK1 levels was measured with a 530-nm bandpass filter. The PI signal corresponding to the DNA content was measured with a 585-nm bandpass filter in conjunction with a 640-nm longpass filter. The doublet $G_0/G_1$ phase cells that resemble singlet $G_2/M$ phase cells were eliminated by setting a gate on the PI pulse intensity vs the PI pulse width blotting field.

### Dot-blot analysis

Cell lysates in 0.01 M Tris-HCl (pH 7.2) and 0.15 M sodium chloride (Tris buffered saline; TBS) were prepared from logarithmic phase cells (3 days after subculture) using an ultra-sonicator. After centrifugation at 3000 ***g*** for 10 min, the supernatants were collected and the total protein concentration was determined using a Bio-Rad Protein Assay kit (Nippon Bio-Rad Laboratory, Tokyo, Japan) following the standard procedure. The protein concentration was adjusted to 100 μg ml$^{-1}$, and serially diluted samples were fixed on a nitrocellulose membrane using a Bio-Dot Microfiltration Apparatus (Bio-Rad). The membrane was then air dried and soaked in the blocking solution (50 mM Tris, 2% BSA, 0.25% gelatin, 0.154 M sodium chloride) for 3 h. The HuMAb SK1 at 5 μg ml$^{-1}$ in 0.5% BSA/TBS was then added and incubated at room temperature for 1 h. It was washed again and incubated with HRP-conjugated goat anti-human IgM (Jackson) for 30 min. After washing, the reactivity was visualized with a peroxidase substrate kit (Bio-Rad)

### SDS-PAGE and Western blot

Cell lysates prepared with an ultra sonicator were mixed with a sample buffer (final concentration: 0.06 M Tris-HCl, 2% SDS, 10% glycerol) and heated at 95°C for 4 min. Acrylamide gel electrophoresis was performed with a Bio-Rad mini-Protean II slab gel apparatus using precast 4–15% linear gradient gels (Bio-Rad) under non-reducing conditions. Separated proteins were then transferred onto a nitrocellulose membrane using a Bio-Rad mini trans-blot apparatus. The blotted filters were stained with HuMAb SK1 as described above.

### RESULTS

### Colon carcinoma reactive antibody

A total of 1036 immunoglobulin-secreting hybridomas were generated from the 18 lymph nodes obtained from six Dukes' B colon carcinoma patients. The cell EIA and the immunocytochemical stainings revealed that 16 antibody-containing supernatants were reactive to colon carcinoma cells and such hybridomas were generated by each of the patients investigated. However, the immunocytochemical stainings of a panel of cultured cell lines with these antibodies showed that most of the selected HuMAbs were not specific to carcinoma but were multi-reactive, even to non-malignant cells (Koda et al, 1990). Among these HuMAbs, SK1 showed the most restricted specificity to carcinoma cell lines (Chang et al, 1993). This carcinoma-preferred reactivity was confirmed by immunohistochemical study in which SK1 reacted mainly with malignant tissues originating in the epithelium (Table 1; Figure 1). In many of the cases the observed immunohistochemical staining was heterogeneous, as clearly seen in Figure 1B and C.

Overall, 107 out of 109 colorectal cancer cases scored as Ag-SK1 positive, as were 10 out of 11 stomach cancers and four out of four pancreatic cancers (Table 1). Breast cancer scored positive in five out of eight cases, representing approximately 62%. Only one case out of 30 normal colorectal mucosa tissues tested scored as Ag-SK1 positive. Weak or trace stainings were observed with liver (two out of seven), pancreas (two out of four) and kidney (one out of four) tissues. All the rest were negative. The overall reactivity pattern was clearly adenocarcinoma-restricted.

### Partial Ag-SK1 analysis

The HuMAb SK1 lost most reactivity to target cells treated with neuraminidase, trypsin or periodate (data not shown), suggesting that the antigen nature of Ag-SK1 was a glycoprotein. The approximate molecular weight of Ag-SK1 was a two-chain structure of 42–46 kDa, as seen by SDS-PAGE with non-reducing conditions, followed by Western blot (Figure 2). Normal colon mucosa of ulcerative colitis and fibroblast cells were negative for this two-chain structure whereas both primary colorectal carcinoma tissue and a colorectal cancer that metastasized to the liver were Ag-SK1 positive (Figure 2).

### Ag-SK1 expression on cancer cells

Analysis of colonies of HT-29 cells, a carcinoma of the colon, indicated that Ag-SK1 expression was regulated by either cell

**Table 1** Immunohistochemical staining of human tissues with HuMAb SK1

| | Number of tissues | | |
|---|---|---|---|
| **Tissues** | **Ag-SK1(+)** | **Ag-SK1(trace)** | **Ag-SK1(–)** |
| Colorectal cancer | 107 | 2 | 0 |
| Colorectal mucosa | 1 | 3 | 26 |
| Stomach cancer | 10 | 1 | 0 |
| Stomach mucosa | 0 | 0 | 11 |
| Pancreatic cancer | 4 | 0 | 0 |
| Pancreas | 0 | 2 | 2 |
| Liver | 0 | 2 | 5 |
| Lung cancer | 2 | 0 | 0 |
| Lung | 0 | 0 | 4 |
| Kidney | 0 | 1 | 4 |
| Brain | 0 | 0 | 4 |
| Melanoma | 0 | 0 | 6 |
| Sarcoma | 0 | 0 | 4 |
| Breast cancer | 5 | 0 | 3 |
| Prostate | 0 | 0 | 3 |
| Skin | 0 | 0 | 3 |
| Oesophagus | 0 | 0 | 3 |
| Bladder | 0 | 0 | 3 |
| Testis | 0 | 0 | 3 |
| Ovary | 0 | 0 | 3 |
| Uterus | 0 | 0 | 3 |
| Spleen | 0 | 0 | 3 |
| Thyroid | 0 | 0 | 3 |
| Heart | 0 | 0 | 3 |
| Bone marrow | 0 | 0 | 3 |
| Eye | 0 | 0 | 3 |
| Nerve | 0 | 0 | 4 |
| Skeletal muscle | 0 | 0 | 6 |
| Salivary gland | 0 | 0 | 1 |

© *Cancer Research Campaign 1998*

**Figure 1** Immunohistochemical staining of colon carcinoma tissues with HuMAb SK1. Four examples (**A–D**) are shown. In many of the cases the observed staining was heterogeneous, as clearly seen in **B** and **C**. Scale bar = 100 μm proliferation or cell density. This is shown in Figure 3. Figure 3A shows the degree on immunostaining by individual cells, and 3B shows a small colony. The outer peripheral region, typically a three- to five-cell-thick layer, of a larger colony of cells (Figure 3C and D) shows good immunostaining, whereas the cells in the centre of the colony are poorly, if at all, stained. The outer peripheral cells of the colony are actively growing, moving outward and therefore expressing Ag-SK1. On the other hand, the inferior cells of the colony are neither growing nor moving, and are Ag-SK1 negative.

To further understand the proliferative nature of Ag-SK1, we analysed the expression of the antigen on Calu-1 cells, a carcinoma of the lung, each day as the culture expanded. These data are shown in Figure 4. Calu-1 cells were seeded at low density (Figure 4, 1D) and allowed to expand until the cultures were greater than 90% confluent, typically by days 6–7. As can be seen, the antigen is diffuse and spread throughout the cell in very low-density cultures. By day 3, the antigen has 'rounded up' and can be seen indenting the nucleus. High-density cultures were Ag-SK1 negative by immunostaining. Similar findings were also observed in PANC-1, pancreatic cancer cells (data not shown). These data suggest that Ag-SK1 is not always expressed on a given carcinoma cell but is metabolized in each cell depending on the circumstances. Two-colour FACS analysis using HuMAb SK1 and PI showed that antigen expression in each cell is independent of the cell cycle status (Figure 5).

### Dot-blot analysis

The amount of Ag-SK1 per extracted cell protein was assessed by dot-blot analysis (Figure 6, Table 2), where the ratio of Ag-SK1-positive cells was determined by FACS analysis (Table 2). We showed that the concentration of Ag-SK1 per protein differs considerably among cell lines, in which SW620, WiDr and LoVo cell lines were considered to express a high amount of Ag-SK1, with the low expression group consisting of Caco-2 and NCI-H716 cell lines. The data shown in Figure 6 were obtained from cells harvested during mid-log growth (between 30% and 50% confluent, usually 3–4 days' culture) when antigen expression was highest.

In FACS analysis, the antigen-positive cells within each cell line were recorded as a discrete subset with more than ten times greater antigen expression in comparison with the negative cells (Figure 5B). The ratio of Ag-SK1-positive cells in each cell line varied in accordance with the harvested time of the cells. However, there was a tendency for cells with high Ag-SK1 concentration in dot-blot analysis to have higher antigen-positive ratios in FACS analysis (Table 2).

 © Cancer Research Campaign 1998

**Figure 2** Western blot analysis of the antigen recognized by HuMAb SK1. Lanes 1–6 were stained with SK1, whereas lanes 8–11 were stained with irrelevant human IgM as a control. Lanes 1 and 8, human fibroblast cell line; lanes 2 and 9, normal colon mucosa of a patient with ulcerative colitis; lane 3, colon carcinoma cell line SW620; lane 4, colon carcinoma cell line LoVo; lanes 5 and 10, primary colon cancer tissue; lanes 6 and 11, liver metastasis tissue of colon cancer; lane 7, molecular weight markers

**Figure 3** Immunocytochemical staining of colony-forming colon carcinoma cell line HT-29. All the cells in the small colony (**A**) were antigen-SK1 positive, whereas in intermediate or large colonies (**B**, **C**), only cells that form the peripheral region of each colony were stained with HuMAb SK1. On a magnified view of **C** (**D**), the intensity of staining differed considerably between inner and outer cells. Scale bar 50 μm in figures **A**, **B** and **D**; 500 μm in **C**

**Figure 4** Successive changes in antigen expression found in Calu-1 lung carcinoma cell line. On 1–7 days (**1D–7D**) after the beginning of subculture, cells were stained with HuMAb SK1. A broadly expressed cytoplasmic antigen, which is seen on **1D** and **2D**, was condensed beside nuclei on **3D** (indicated by arrows). The antigen was dispersed again on **4D**, and was hardly seen on **7D** when cells become more than 90% confluent.Scale bars = 50 μm

**Table 2** Cell invasion capacity and concentration of Ag-SK1

| Cell line[a] | Number of migrated cells (mean ± s.d.) | Concentration of Ag-SK1 assessed by dot-blot analysis[b] | Ag-SK1 positive cells determined by FACS[c] (%) |
|---|---|---|---|
| SW620 | 232 ± 52 | 32× | >90 |
| WiDr | 155 ± 37 | 32× | 40–70 |
| LoVo | 89 ± 23 | 16× | 60–80 |
| LS174T | 93 ± 21 | 8× | 60–80 |
| COLO320DM | 72 ± 15 | 8× | 30–50 |
| HT-29 | 22 ± 13 | 8× | 40–60 |
| NCI-H716 | <10 | 4× | 30–50 |
| Caco-2 | <10 | 2× | 20–40 |

[a]Mid-log phase cells (30–50% confluent) were harvested and prepared for analysis. [b]Data indicate maximum dilution ratio of the extracted protein that can be detected by HuMAb SK1 on dot-blot analysis (see Materials and methods). [c]The positive ratio was calculated with Kolgomolov–Smirnov statistics.

### Invasion capacity of colon carcinoma cell lines

The cell invasion capacity was determined with a microchemotaxis assay, and the data are summarized in Table 2. The number of cells that migrated to the lower chamber was high in SW620 and WiDr cell lines, whereas almost no invaded cells were observed in NCI-H716 and Caco-2 cells. When comparing the cell invasion capacity with the amount of Ag-SK1 in each cell line, there was a tendency for the cell lines with high invasion capacity to have a higher amount of Ag-SK1 (Table 2).

To confirm the hypothesis that Ag-SK1 was an invasion- or a migration-related molecule of colon carcinomas, we next investigated whether the invasion capacity of these cells was inhibited by the addition of HuMAb SK1 into the upper sides of the chemotaxis chambers in a quadruplicate assay. As shown in Figure 7, only a

 *© Cancer Research Campaign 1998*

**Figure 5** Cell cycle-independent expression of the antigen on colon carcinoma cell line, SW620. The vertical line indicates propidium iodide (PI) signal in linear scale corresponding to DNA content of each cell, whereas the horizontal line indicates log fluorescein isothiocyanate (FITC) signal obtained from (**A**) human polyclonal IgM antibody, 5 µg $ml^{-1}$ and (**B**) HuMAb SK1, 5 µg $ml^{-1}$. There was no correlation between DNA content (cell cycle) and the number of Ag-SK1-positive cells

**Figure 6** Dot-blot analysis for determining the antigen concentration in colon carcinoma cell lines. Aliquots of 100 mg of extracted cell protein from each cell line were dotted on the first column. From the second column, serially double-diluted samples were dotted. The membrane was then stained with HuMAb SK1. Cell lines used were: 1, SW620; 2, WiDr; 3, LoVo; 4, LS174T; 5, COLO320DM; 6, HT-29; 7, NCI-H716; 8, Caco-2

limited number of cells migrated to the lower side of the membrane after 36 h of incubation with HuMAb SK1. This inhibition was dose dependent and up to 87% of cell migration was blocked with 1 µg $ml^{-1}$ HuMAb Sk1 (Figure 8).

### DISCUSSION

Data have been accumulating indicating that the overall immune system of cancer-bearing patients is able to recognize autologous tumour antigens specifically. In cellular immunity, the cytotoxic T lymphocyte that specifically targets autologous tumours has been induced in vitro (Barnd et al, 1989; Wright et al, 1989; Topalian et al, 1989; Peoples et al, 1995). In humoral immunity, human monoclonal antibodies against cancers have been primarily generated via hybridoma technology using B lymphocytes from cancer-bearing patients (Yamaguchi et al, 1990; Posner et al, 1991; Hoon et al, 1993; Oka et al, 1994). In spite of the existence of this specific cancer recognition by the patient's immune system, tumours do grow in a physiological environment, implying that the humoral response was an insufficient contribution to the anti-tumour effect. The basic strategy of cancer biotherapy is to magnify or to modify the innate anti-tumour immune system using a variety of 'bioweapons' such as cytokines (Rosenberg et al, 1989; Weiner et al, 1991; Dillman et al, 1993; Ernstoff 1994), gene transfer (Porgador et al, 1993; Tohmatsu et al, 1993; Iwanuma et al, 1995), adoptive transfer of activated killer cells (Rosenberg et al, 1986; Andreesen et al, 1990; Keilholz et al, 1994) and cancer vaccinations (Elliott et al, 1993; Hoover et al, 1993; Houghton, 1995). So far, the majority of these approaches have involved cellular immunity, whereas human antibodies have been used in in vivo diagnosis of malignancies only (Ditzel et al, 1993; Chaudhuri et al, 1994; Ditzel et al, 1994).

The expected anti-tumour effects of human monoclonal antibodies could be listed as follows: (1) to bind the functional molecule in the metastasizing process of cancer cells, such as invasion, destroying basal membrane, or implantation in distant organs, thereby blocking the cancer metastases or expansions; (2) to induce antibody-dependent cell-mediated cytotoxicity (ADCC) or complement dependent cytolysis (CDC) against cancer cells; and (3) to induce anti-idiotypic antibody against the administered HuMAbs. This second antibody may mimic the tumour-associated antigen(s) and offer an opportunity to induce a cell-mediated immune response specific to tumour cells (Ferrone 1993; Fargerberg et al, 1995).

The current data indicate that the antigen that is recognized by HuMAb SK1 is a two-chain molecule with approximate molecular weight of 42–46 kDa, as it appears from the two most prominent bands (Figure 2). Tissue specificity analysis showed that HuMAb SK1 preferentially stained adenocarcinoma tissues with minimal to negative staining among non-epithelial tumours and normal epithelium (Table 1). The antigen-positive cells within each colon cancer cell line seemed to represent a discrete subset with different levels of Ag expression, judging from FACS analysis (Figure 5) and from the immunocytochemical staining of HT-29 (Figure 3). There was a variety of Ag-positive ratios observed, depending upon the harvested time of the cells (FACS data from Table 2). Furthermore, there were variations in antigen location and inten-

© Cancer Research Campaign 1998

**Figure 7** Microscopic features of upper and lower sides of the membrane in the chemotaxis chamber. LoVo colon cancer cells migrated to the lower side of the membrane after 36 h of incubation (left arrow). This cell migration was almost completely inhibited by the addition of HuMAb SK1 in the upper chamber (right). Scale bars = 200 μm

**Figure 8** Dose-dependent inhibition of cell migration with HuMAb SK1 added in the upper chemotaxis chamber. The vertical line indicates a number of migrated cells to the lower side of the membrane per $10^5$ cells. The horizontal line indicates concentration of antibodies. No inhibition was observed with irrelevant human IgM MAb. Cell line used was LoVo colon cancer cell line. ■, SK1; ●, IgM

sity in Calu-1 and PANC-1 cells (Figure 4). These data suggest that the antigen is not always expressed on a given cell but is possibly metabolized in each cell depending on the circumstances. A dot-blot analysis and FACS analysis showed that the invasion capacity of eight colon carcinoma cell lines correlates with the Ag-SK1 concentration of each cell line and with the ratio of Ag-SK1-positive cells (Figure 6, Table 2). In addition, cell migration was inhibited by the addition of HuMAb SK1 in a dose-dependent manner. Taken together, these data suggest that HuMAb SK1 recognizes an antigen related to colon carcinoma cell invasion that appears to be not only metabolized according to physiological circumstances but is also expressed on highly invasive cell populations.

Although SK1 did not cross-block with mouse monoclonal antibodies directed to the known invasion- or metastasis-related molecule such as E-cadherin, sialyl Tn, sialyl $Le^x$, sialyl $Le^a$, vitronectin receptor alpha-chain, integrin beta 1- or beta 3-chains (data not shown), the possibility that SK1 recognizes an epitope that contains some of these molecules still remains, as the mouse and human MAb might recognize different epitopes on the same molecule. This is an issue to be analysed further.

In the current study, we evaluated 1036 HuMAbs derived from 18 regional lymph nodes of six Dukes' B colon cancer patients. Among them, a high reaction specificity against cancer cells was seen in only one HuMAb, SK1. Although we used the lymphocytes derived from the nearest lymphoid system to the tumour, the appearance frequency of specific HuMAbs was unexpectedly small, suggesting that most of the patients may not raise a humoral immune response to autologous tumours, otherwise the B-cell population that is primed to secrete a specific antibody to tumours is small in number or is not fully activated to be fused (Koda et al, 1990).

The inhibition of in vitro colon cancer invasiveness by HuMAb SK1 required as much as 1 μg $ml^{-1}$ of antibody concentration (Figure 8), which is unlikely to be secreted by such small cell

 © Cancer Research Campaign 1998

populations of tumour-specific B lymphocytes in regional lymph nodes. This suggests that human natural antibodies to carcinoma, even when they exist, may not play an anti-cancer role effectively under physiological conditions.

A HuMAb SK1 is considered to be an augmentation of the natural humoral immune response of cancer-bearing patients. It may encourage successful cancer biotherapy using such HuMAbs that exert a direct action upon cancer invasion and metastases. Although the appearance of SK1 was low in frequency (1 out of 1036), its existence does suggest that there may be others also specific to cancer. The task then is to develop appropriate search protocols to prove effectively the entire human immune response repertoire and identify those sequences that identify cancer-specific antigens.

The availability of immunoreagents such as SK1 will not only provide new insights into the working of the anti-cancer human immune response but also give the oncologist clinically useful antibodies for better diagnosis and therapy.

### ABBREVIATIONS

Ag, antigen; HuMAb, human monoclonal antibody; kDa, kilodalton; HRP, horseradish peroxidase; EIA, enzyme immunoassay; PI, propidium iodide; SDS-PAGE, sodium dodecyl sulphate-polyacrylamide gel electrophoresis; FACS, fluorescence-activated cell sorter.

### ACKNOWLEDGEMENT

This work was partially supported by a Grant-in-aid for Scientific Research from the Japanese Ministry of Education (no. 09770930).

### REFERENCES

Andreesen R, Scheibenbogen C, Brugger W, Krause S, Meerpohl HG, Leser HG, Engler H and Lohr GW (1990) Adoptive transfer of tumor cytotoxic macrophages generated in vitro from circulation blood monocytes: a new approach to cancer immunotherapy. *Cancer Res* **50**: 7450–7456

Aznavoorian S, Liotta LA and Kupchik HZ (1990) Characteristics of invasive and noninvasive human colorectal adenocarcinoma cells. *J Natl Cancer Inst* **82**: 1485–1493

Barnd DL, Lan MS, Metzgar RS and Finn OJ (1989) Specific major histocompatibility complex-unrestricted recognition of tumor-associated mucins by human cytotoxic T cells. *Proc Natl Acad Sci USA* **86**: 7159–7163

Borrebaeck CAK (1987) Human monoclonal antibodies produced from primary in vitro immunized leucine methyl ester-treated peripheral blood lymphocytes. In *In Vitro Immunization in Hybridoma Technology*, Borrebaeck CAK (ed.) pp. 209–229. Elsevier: The Netherlands

Borrebaeck CAK, Danielsson L and Möller SA (1988) Human monoclonal antibodies by primary in vitro immunization of peripheral blood lymphocytes. *Proc Natl Acad Sci USA* **85**: 3995–3999

Chang HR, Koda K, Chang S and Baird S (1993) Ag-SK1, a novel carcinoma associated antigen. *Cancer Res* **53**: 1122–1127

Chaudhuri TR, Zinn KR, Morris JS, McDonald GA, Llorens AS, and Chaudhuri TK (1994) Detection of ovarian cancer by $^{198}$Au-labeled human monoclonal antibody. *Cancer* **73**: 878–883

Cole SPC, Campling BG, Louwman IH, Kozbor D and Roder JC (1984) A strategy for the production of human monoclonal antibodies reactive with lung tumor cell lines. *Cancer Res* **44**: 2750–2753

Dillman RO, Church C, Oldham RK, West WH, Schwartzberg L and Birch R (1993) Inpatient continuous-infusion interleukin-2 in 788 patients with cancer. The National Biotherapy Study Group experience. *Cancer* **71**: 2358–2370

Ditzel H, Rasmussen JW, Erb K, Borup-Christensen P, Titlestad I, Lassen E, Fenger C, Kronborg O and Jensenius JC (1993) Tumour detection with $^{131}$I-labeled human monoclonal antibody COU-1 in patients with suspected colorectal carcinoma. *Cancer Res* **53**: 5920–5928

Ditzel H, Rasmussen JW, Borup-Christensen P, Erb K and Jensenius JC (1994) Immunoscintigraphy of colon cancers with the human monoclonal antibody COU-1. *Cancer* **73**: 858–863

Elliott GT, McLeod RA, Perez J and Von Eschen KB (1993) Interim results of a phase II multicenter clinical trial evaluating the activity of a therapeutic allogeneic melanoma vaccine (theraccin) in the treatment of disseminated malignant melanoma. *Semin Surg Oncol* **9**: 264–272

Ernstoff MS (1994) Combination cytokine therapy in cancer. In *Tumor Immunology and Cancer Therapy*, Goldfarb RH, Whiteside TL (eds) pp. 273–279. Marcel Dekker: New York

Fagerberg J, Hjelm AL, Ragnhammar P, Frodin JE, Wigzell H and Mellstedt H (1995) Tumor regression in monoclonal antibody-treated patients correlates with the presence of anti-idiotype-reactive T lymphocytes. *Cancer Res* **55**: 1824–1827

Ferrone S (1993) Human tumor associated antigen mimicry by anti-idiotypic antibodies. Immunogenicity and clinical trials in patients with solid tumors. *Ann NY Acad Sci* **690**: 214–224

Glassy MC (1987) Immortalization of human lymphocytes from a tumor-involved lymph node. *Cancer Res* **47**: 5181–5188

Glassy MC (1989) Generation of UC-729-6 and SHFP-1 derived human hybridomas and their growth in serum-free media. *J Tissue Culture Methods* **12**: 85–89

Glassy MC and Surh CD (1985) Immunodetection of cell-bound antigens using both mouse and human monoclonal antibodies. *J Immunol Methods* **81**: 115–122

Glassy MC and McKnight ME (1994) Pharming the human lymph node. *Exp Opin Invest Drugs* **3**: 1057–1060

Glassy MC, Handley HH, Hagiwara H and Royston I (1983) UC 729-6, a human lymphoblastoid B-cell line useful for generating antibody-secreting human–human hybridomas. *Proc Natl Acad Sci USA* **80**: 6327–6331

Hoon DSB, Wang Y, Sze L, Kanda H, Watanabe T, Morrison SL, Morton DL and Irie RF (1993) Molecular cloning of a human monoclonal antibody reactive to ganglioside GM3 antigen on human cancer. *Cancer Res* **53**: 5244–5250

Hoover HCJr, Brandhorst JS, Peters LC, Surdyke MG, Takeshita Y, Madariaga J, Muenz LR, and Hanna Jr MG (1993) Adjuvant active specific immunotherapy for human colorectal cancer: 6.5-year median follow-up of a phase III prospectively randomized trial. *J Clin Oncol* **11**: 390–399

Houghton AN (1995) On course for a cancer vaccine. *Lancet* **345**: 1384–1385

Inoue H, Hirohashi S, Shimosato Y, Enjoji M, Clausen H and Hakomori S-I (1989) Establishment of an anti-A human monoclonal antibody from a blood group A lung cancer patient: evidence of autoimmune response to difucosylated type-2 chain A. *Eur J Immunol* **19**: 2197–2203

Iwanuma Y, Kato K, Yagita H and Okumura K (1995) Induction of tumor-specific cytotoxic T lymphocytes and natural killer cells by tumor cells transfected with the interleukin-2 gene. *Cancer Immunol Immunother* **40**: 17–23

Keilholz U, Scheibenbogen C, Brado M, Georgi P, Maclachlan D, Brado B and Hunstein W (1994) Regional adoptive immunotherapy with interleukin-2 (IL-2) and lymphokine-activated killer (LAK) cells for liver metastasis. *Eur J Cancer* **30A**: 103–105

Kjeldsen TB, Rasmussen BB, Rose C and Zeuthen J (1988) Human–human hybridomas and human monoclonal antibodies obtained by fusion of lymph node lymphocytes from breast cancer patients. *Cancer Res* **48**: 3208–3214

Koda K and Glassy MC (1990) In vitro immunization for the production of human monoclonal antibodies. *Hum Antibod Hybridomas* **1**: 15–22

Koda K, Glassy MC and Chang HR (1990) Generation of human monoclonal antibodies against colon cancer. *Arch Surg* **125**: 1591–1597

McKnight ME and Glassy MC (1995) The autoimmunity of cancer as applied to drug development. *Exp Opin Invest Drugs* **4**: 657–661

Miyake M, Taki T, Kannagi R and Hitomi S (1992) First establishment of a human monoclonal antibody directed to sulfated glycosphingolipids $S_{M4S\text{-}Gal}$ and $S_{M4g}$, from a patient with lung cancer. *Cancer Res* **52**: 2292–2297

Oka T, Kikumoto Y, Itakura, K Morton DL and Irie RF (1994) Human monoclonal antibody identified an immunoreactive tetrapeptide sequence (Lys-Tyr-Gln-Ile) in M(r) 43,000 protein of human melanoma. *Cancer Res* **54**: 3511–3515

Peoples GE, Goedegebuure PS, Smith R, Linehan DC, Yasho I and Eberlein TJ (1995) Breast and ovarian cancer-specific cytotoxic T lymphocytes recognize the same HER2/neu-derived peptide. *Proc Natl Acad Sci USA* **92**: 432–436

Pfaff M, O'Connor R, Vollmer HP and Müller-Hermelink HK (1990) Human monoclonal antibody against a tissue polypeptide antigen-related protein from a patient with a signet-ring cell carcinoma of the stomach. *Cancer Res* **50**: 5192–5198

Porgador A, Bannerji R, Watanabe Y, Feldman M, Gilboa E and Eisenbach L (1993) Antimetastatic vaccination of tumor-bearing mice with two types of INF-gamma gene-inserted tumor cells. *J Immunol* **150**: 1458–1470

© Cancer Research Campaign 1998

Posner MR. Elboim HS. Tumber MB. Wiest PM and Tibbetts LM (1991) An IgG human monoclonal antibody reactive with a surface membrane antigen expressed on malignant breast cancer cells. *Hum Antibod Hybridomas* **2**: 74–83

Rosenberg SA. Lotze MT. Muul LM. Leitman S. Chang AE. Vetto JT. Seipp CA and Simpson C (1986) A new approach to the therapy of cancer based on the systemic administration of autologous lymphokine-activated killer (LAK) cells and recombinant interleukin-2. *Surgery* **100**: 262–272

Rosenberg SA. Lotze MT. Yang JC. Linehan WM. Seipp C. Calabro S. Karp SE. Sherry RM. Steinberg S and White DE (1989) Combination therapy with interleukin-2 and alpha-interferon for the treatment of patients with advanced cancer. *J Clin Oncol* **7**: 1863–1874

Shi S-R. Key ME and Kalra KL (1991) Antigen retrieval in formalin-fixed. paraffin-embedded tissues: an enhancement method for immunohistochemical staining based on microwave oven heating of tissue sections. *J Histochem Cytochem* **39**: 741–748

Tohmatsu A. Okino T. Stabach P. Padula SJ. Ergin MT and Mukherji B (1993) Analysis of cytolytic effector cell response in vitro against autologous human tumor cells genetically altered to synthesize interleukin-2. *Immunol Lett* **35**: 51–57

Topalian SL. Solomon D and Rosenberg SA (1989) Tumor-specific cytolysis by lymphocytes infiltrating human melanomas. *J Immunol* **142**: 3714–3725

van den Berg FM. Baas IO. Polak MM and Offerhaus GJ (1993) Detection of p53 overexpression in routinely paraffin-embedded tissue of human carcinomas using a novel target unmasking fluid. *Am J Pathol* **142**: 381–385

Weiner LM. Padavic-Shaller K. Kitson J. Watts P. Krigel RL and Liwin S (1991) Phase I evaluation of combination therapy with interleukin 2 and gamma-interferon. *Cancer Res* **51**: 3910–3918

Wright A. Lee JE. Link MP. Smith SD. Carroll W. Levy R. Clayberger C and Krensky AM (1989) Cytotoxic T lymphocytes specific for self tumor immunoglobulin express T cell receptor delta chain. *J Exp Med* **169**: 1557–1564

Yamaguchi H. Furukawa K. Fortunato SR. Livingston PO. Lloyd KO. Oettgen HF and Old LJ (1990) Human monoclonal antibody with dual GM2/GD2 specificity derived from an immunized melanoma patient. *Proc Natl Acad Sci USA* **87**: 3333–3337

© Cancer Research Campaign 1998